# A New Perspective on Osteogenesis Imperfecta: From Cellular Mechanisms to the Systemic Impact of Collagen Dysfunction

**DOI:** 10.3390/ijms27020745

**Published:** 2026-01-12

**Authors:** Emma Lugli, Ludovica Gaiaschi, Maria Grazia Bottone, Fabrizio De Luca

**Affiliations:** Department of Biology and Biotechnology, University of Pavia, 27100 Pavia, Italy; emma.lugli01@universitadipavia.it (E.L.); ludovica.gaiaschi@unipv.it (L.G.); mariagrazia.bottone@unipv.it (M.G.B.)

**Keywords:** osteogenesis imperfecta, collagen dysfunction, in vivo models, redox imbalance, intracellular stress, ER stress, mitochondrial stress, cell death, autophagy, systemic inflammation

## Abstract

Osteogenesis imperfecta (OI) is a rare genetic disease caused by mutations in collagen type I, leading to defective protein folding and an impaired extracellular matrix structure and remodelling. Beyond skeletal fragility, these molecular defects trigger a network of intracellular stress responses with multiorgan implications: the accumulation of misfolded collagen can induce persistent endoplasmic reticulum stress, which can in turn compromise mitochondrial function and autophagy or lead to cell death activation, and it can even promote widespread redox imbalance and inflammation. The interplay between intracellular stress, widespread oxidative damage and inflammation not only underlies cellular dysfunction but also the multisystemic manifestations of osteogenesis imperfecta. Targeting these interconnected pathways may result in new insights for a better understanding of OI and possibly offer novel therapeutic strategies designed to restore proteostasis and improve cell homeostasis and overall patient outcomes, highlighting the need for an integrated understanding of the cellular and molecular mechanisms involved in the pathogenesis of this disease and their translation into patient-centred therapeutic interventions.

## 1. Introduction

Historically referred to as brittle bone disease, osteogenesis imperfecta (OI) is a rare genetic disease with an estimated prevalence of 1 in every 15,000–20,000 live births worldwide. Characterised by both clinical and genetical heterogeneity, OI consists of a broad range of systemic connective tissue disorders caused by alterations in the synthesis, modification, folding, trafficking and processing of collagen type I (COL1), a key component of the extracellular matrix (ECM) [1]. The earliest description of OI as a clinical condition dates back to the 1840s, when W. Vrolik coined the term ‘osteogenesis imperfecta’, from the Latin ‘imperfect bone formation’, to describe skeletal dysplasias that were not acquired postnatally [2]. In 1979, D. Sillence then proposed the first practical nosology, dividing OI into four groups (types I–IV) based on patients’ phenotypic and radiographic features, as well as on the severity and inheritance pattern [3]. At present, Sillence’s grouping is still used as a clinical classification system, yet it fails to account for the broad phenotypic variability and overlap between groups: in the last few decades, technical advances in genomic sequencing have enabled better molecular characterisation, shedding light on OI’s pathophysiological mechanisms and necessitating a refined genetic classification system. Roughly 85–90% of OI cases are caused by autosomal dominant mutations within the genes encoding the COL1 α1 and α2 chains (*COL1A1* and *COL1A2*, respectively), first discovered in the early 1980s. Since then, several other pathogenetic variants, mostly with autosomal or X-linked recessive inheritance, have been identified in genes involved in collagen posttranslational modification and processing, trafficking and osteoblast differentiation/mineralisation [4]. Therefore, as the number of causative genes has grown over the years, the initial Sillence classification has been continuously revised and expanded to achieve more accurate categorisation and to better reflect the disease’s heterogeneity. Recently, an updated dyadic nosology was introduced, linking clinical phenotypes and underlying genetic variants, thereby providing a refined grouping for the original Sillence types [5]. In addition, in 2017, a functional/metabolic classification system was proposed wherein genes are grouped based on their shared mechanisms in order to accurately encompass both genetic and clinical features (Table 1) [6].

Despite the marked genetic heterogeneity, the disease is well known for its skeletal implications, which represent a unifying feature across patients: bone fragility, increased susceptibility to fractures, malformations (e.g., chest wall and long bone deformities), macrocephaly, scoliosis and kyphosis, joint issues and generalised osteopenia. Nevertheless, OI clinical outcomes cover a wide range of extraskeletal manifestations, including muscle weakness, fatigue, cardiorespiratory complications, renal and vascular impairments, hearing loss, blue sclerae, dentinogenesis imperfecta and varying degrees of neurocranial malformation and neurologic implications [7]. Emerging evidence also indicates systemic inflammation and immune system activation as part of the pathology. Moreover, recent cohort studies have shown that OI patients display increased susceptibility to glaucoma and gastrointestinal inflammatory diseases, corroborating the broader multisystemic implications [8,9,10,11].

Owing to its well-recognised bone phenotype, OI diagnosis builds upon the skeletal features observed in patients: normally occurring during childhood, the diagnostic workup is based on clinical observations, family and medical history, physical evaluations and radiographic examinations. Diagnosis could also occur before birth as prenatal ultrasounds can show alterations indicative of OI (e.g., reduced bone mass density) or specific malformations; biochemical analyses in chorionic villus cells are also effectively employed to diagnose qualitative OI. Since several OI symptoms are shared with other skeletal or bone fragility disorders, genetic testing is often required when clinical and physical evaluation is not sufficient or to confirm the diagnosis and estimate prognosis. According to the European Molecular Genetics Quality Network’s best practice guidelines, the current first-line approach relies on genetic sequencing for *COL1A1* and *COL1A2* to identify causative variants most commonly found in OI patients. High-throughput sequencing can also be used to scan for all known genes associated with OI, and preferred samples include blood, saliva or fibroblasts, as well as choroid villus samples, amniocytes or blastocysts for postnatal and prenatal/preimplantation diagnosis, respectively [12,13].

Depending on its severity, OI can result in highly debilitating outcomes that severely affect patients’ lives. To date, treatment still remains largely supportive and symptomatic: multidisciplinary approaches are currently the best options for disease management but almost exclusively focus on OI’s skeletal implications, namely bone fragility and muscle weakness. Currently, the standard pharmacological intervention relies on bisphosphonates (BP), with either intravenous or oral administration, and also involves calcium and vitamin D supplementation. BP therapy begins during infancy and continues through growth, aiming to reduce bone reabsorption in order to increase the bone mineral density (BMD), thereby preventing frequent fractures [14]. Recent clinical trials have also demonstrated that bone anabolic agents (e.g., sclerostin inhibitors and TGF beta inhibitors) effectively improve bone health and fracture risks in adults [15,16]. Alongside BMD-increasing drugs, orthopaedic interventions (involving physiotherapy, orthotic supports and orthopaedic surgery) are employed in OI management to increase physical function and motility, while non-steroidal anti-inflammatory drugs are employed to reduce pain and improve patients’ overall quality of life (QoL) [17]. Other approaches designed to treat bone fragility include stem cell therapy; bone marrow transplantation with adult stem cells (mesenchymal stem cells (MSCs) and hematopoietic stem cells (HSCs)) has shown promising results in OI murine models as well as in child patients, improving bone matrix production and even preventing perinatal mortality [18,19,20]. Additionally, novel, transformative strategies in OI involve gene therapy to treat—or even prevent—OI symptomatology; advances in gene editing technologies have enabled the correction of *COL1A1* and *COL1A2* mutations. Gene silencing has also proven successful in preclinical studies: siRNAs and antisense oligonucleotides directed against mutant *COL1* have shown promise in reducing defective collagen synthesis [21,22,23].

**Figure 1:**
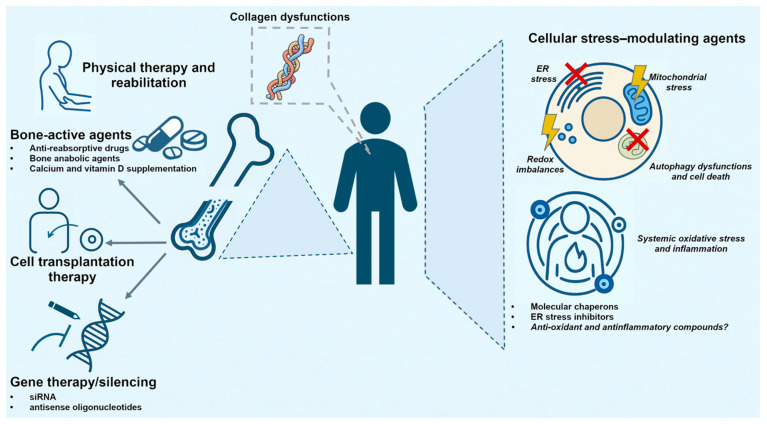
**Schematic overview of clinical management and novel therapies in osteogenesis imperfecta.** The figure summarises the major targets employed in OI. Current therapeutic options are largely symptomatic, primarily directed towards bone fragility and related pain: standard pharmacological intervention includes bone-active agents, in combination with rehabilitation and physical therapy; in addition, experimental approaches targeting OI skeletal manifestations include stem cell-based and gene therapy. Parallelly to bone-targeted interventions, emerging approaches aim to mitigate intracellular stress, which can also reduce systemic redox imbalance and inflammation.

Mutant procollagen can misfold in the endoplasmic reticulum (ER) and lead to COL1 retention, ultimately causing intracellular perturbations that include ER stress, mitochondrial dysfunction and autophagy upregulation [24,25,26]. Therefore, therapies targeting intracellular stress pathways have been emerging as promising strategies in OI management. Preclinical studies have demonstrated that the administration of the proteasome inhibitor Bortezomid significantly improves osteoblast differentiation and enhances bone parameters by reducing autophagy in MSCs. Additionally, treatment with chaperones has been shown to alleviate ER dilation, restore proteostasis, enhance autophagy and collagen secretion and reduce apoptosis, thereby supporting the idea that ER stress represents a key mechanism linking collagen biosynthesis defects to the loss of intracellular homeostasis in OI, both in vivo and in vitro (Figure 1) [27,28,29,30].

In this context, the limited efficacy of current therapeutic options has prompted increasing efforts to dissect the molecular/cellular mechanisms underlying OI’s pathogenesis and systemic effects. A deeper understanding of these processes has become essential to identify rational therapeutic targets; thus, appropriate experimental models are needed. Indeed, a large number of different animal models have been established to more accurately reflect the molecular and cellular mechanisms driving OI symptoms and to promote drug discovery [31]. Several murine models, including both spontaneous and transgenic lines, have been developed to reproduce the key features of the disease. Transgenic mouse models widely employed in preclinical research include (i) oim mice, with a deletion in the *COL1A2* gene (at position 3983) and characterised by frequent fractures and osteopenia; (ii) heterozygous brittle mice (Brtl/+), carrying a specific *COL1A1* substitution, with well-reproduced dominant OI features including reduced bone mass density, impaired collagen folding and overall ECM alterations; (iii) Jrt heterozygous mice; (iv) Aga2 mice, where a frameshift mutation in the *COL1A1* gene causes procollagen α1-chain retention, leading to a disorganised collagen network; (v) Crtap mice, characterised by defects in collagen posttranslational modification and altered fibril formation; and (vi) IFITM5 transgenic mice, with prominent skeletal manifestations due to abnormal osteoblastic differentiation [32]. Besides the well-studied mammalian systems, small-sized vertebrate models have started to gain prominence as well. In particular, due to its genetic similarity to humans and rapid development, the zebrafish (*Dario renio*) has emerged as a valuable model that well recapitulates both the skeletal and extraskeletal features of OI; specifically, the chihuahua (Chi/+) COL1A1 mutant is one of the most extensively studied zebrafish models and is frequently employed in OI drug screening [33]. Complementary to in vivo approaches, patient-derived in vitro models provide a human-specific tool to study OI, enabling the direct analysis of collagen processing defects and allowing the thorough investigation of the response to therapeutic strategies. MSCs developed from patients’ fibroblasts offer unlimited proliferation and a multilineage differentiation capacity, allowing long-term culture, drug screening and studies on disease mechanisms (Table 2) [34].

**Table 2:** Summary of experimental models most commonly employed to study OI; for each model, the type (in vivo or in vitro), species, affected gene, type of mutation and defining defects are listed.

Model	Type	Species/System	Gene	Mutation	Effects
**OIM**	In vivo	*Mus musculus*	*COL1A2*	Deletion	Primary COL1 defects
**Brittle (Brtl/+)**	In vivo	*Mus musculus*	*COL1A1*	Substitution	Primary COL1 defects
**Jrtl**	In vivo	*Mus musculus*	*COL1A1*	Substitution	Primary COL1 defects
**Aga2**	In vivo	*Mus musculus*	*COL1A1*	Substitution	Primary COL1 defects
**Crtap**	In vivo	*Mus musculus; Dario renio*	*CRTAP*	Knock-out	COL1 modification defects
**IFITM5**	In vivo	*Mus musculus*	*IFITM5*	Substitution	Defects in osteoprogenitor differentiation
**Chihuahua (Chi/+)**	In vivo	*Dario renio*	*COL1A1*	Substitution	Primary COL1 defects
**OI iPSCs**	In vitro	Human-/animal-derived iPSCs	Patient-/model-specific		

Since existing experimental models and therapies only partly address osteogenesis imperfecta’s features and symptoms, increasing attention has been paid to the study of cellular mechanisms and strategies targeting intracellular stress.

The aim of this review is to summarise current knowledge regarding OI’s cellular drivers, shedding light on cytological alterations involved in this pathology and revealing those that still require further investigation, in order to advance our mechanistic understanding of OI’s pathophysiology, improve the management of patients and possibly guide the development of newly targeted therapeutic approaches.

## 2. Intracellular Stress and Homeostatic Imbalance in OI

The unifying pathogenic feature of osteogenesis imperfecta is the presence of COL1 dysfunction, thus classifying the disease as a collagen-related disorder [4]. Collagens are major constituents of the extracellular matrix, a complex and highly organised network that provides structure and support, modulates cell signalling within tissues and is involved in the regulation of cell growth and differentiation, ultimately driving tissue development [35]. Specifically, COL1 is a heterotrimeric protein, constituted by three α chains (two α1 and one α2, encoded by the *COL1A1* and *COL1A2* genes, respectively) and is synthesised as a procollagen molecule in the ER; then, it is transported to the Golgi apparatus, where it is assembled into mature collagen type I fibrils. In the ER, procollagen undergoes several posttranslational modifications involving specific proteins crucial for proper COL1 fibril assembly, including (i) the removal of N-terminal signals and peptide stabilisation; (ii) the hydroxylation of lysine and proline residues by hydroxylation enzymes; (iii) the glycosylation of hydroxyl groups by galactosyl- and glycosyl-transferases; and (iv) modified pro-α chains’ folding and assembly into procollagen molecules [36]. Most OI cases are caused by qualitative or quantitative defects in COL1 synthesis: the vast majority of autosomal-dominant pathogenic variants (roughly 85–90%) affect the *COL1A1* and *COL1A2* genes, yet all mechanisms involved in collagen type I biosynthesis are known to be linked to OI pathophysiology, from its synthesis, modification and folding to its secretion into the ECM. Other causative mutations also involve genes responsible for collagen posttranslational modification and processing, trafficking, ossification and mineralisation, as well as osteoblast differentiation [4].

**Figure 2:**
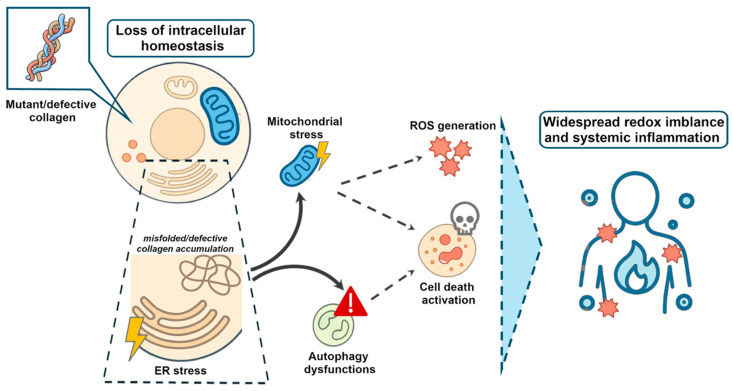
**Loss of intracellular homeostasis and systemic consequences in osteogenesis imperfecta.** The diagram illustrates the cascade of intracellular stress pathways, beginning with altered ER functionality, driven by defective collagen synthesis, to mitochondrial and autophagic dysfunction; increased ER stress leads to altered proteostasis, disrupting the autophagic machinery, and also determines increased mitochondrial stress. As a consequence, elevated ROS production and increased cell death promote widespread oxidative stress/inflammation, contributing to multisystem tissue damage.

Although COL1 dysfunction represents a key feature in OI, the processes linking these defects to systemic manifestations are not fully characterised and may involve the loss of intracellular homeostasis, including ER stress and altered proteostasis, mitochondrial stress and autophagy overload, as well as systemic inflammation and oxidative stress [37] (Figure 2).

### 2.1. ER Stress and Altered Proteostasis

The endoplasmic reticulum is responsible for the synthesis and modification, as well as the folding and quality control, of proteins destined for secretion or membrane insertion [38]. Disruptions affecting the ER compartment have been reported in OI, potentially arising through different mechanisms, which may culminate in the activation of intracellular stress responses and ultimately influence disease pathophysiology. Indeed, mutant COL1, particularly when involving glycine substitution, displays improper and slower folding, leading to increased residence within the ER. This prolongs exposure to modifying enzymes and, consequently, can cause collagen overmodification [30,39]. Alternatively, recessive OI mutations can directly affect collagen modification enzymes and scaffold proteins, including prolyl 3-hydroxylase 1 (P3H1) and cartilage-associated protein (CRTAP), leading to altered COL1 processing [39,40]. Moreover, rare OI cases are even linked to ER proteins such as HSP47 and FKBP10, specific type I procollagen chaperons [41]. Disturbances in the folding machinery may overload and compromise the folding/modification capacity, leading to protein accumulation and impairing endoplasmic reticulum homeostasis [38]. Such a condition is commonly referred to as ‘ER stress’ and can result in the activation of the unfolded protein response (UPR), an adaptive response attempting to counteract the misfolded protein accumulation by inhibiting new protein synthesis and by improving protein degradation [42]. As related to osteogenesis imperfecta, ER dysfunction and UPR activation have been documented both in vitro and in vivo and likely result from mutant/misfolded collagen accumulation, which induces intracellular stress and may lead to downstream apoptosis if unresolved [25,43,44,45]. Electron microscope ultrastructural investigations, in humans and in experimental models, have revealed enlarged ER cisternae, an altered thickness and vacuolisation, consistent with COL1 accumulation, intracellular retention and UPR activation [46,47]. Similarly, osteoblasts and chondrocytes from mice carrying a mutation in the *COL1A2* gene exhibited misfolded collagen accumulation, which ultimately leads to autophagy activation; likewise, in osteoblasts from the Aga2 mouse model, the intracellular accumulation of mutant procollagen I α chains led to the activation of apoptotic events [25,44,48].

In accordance with this evidence regarding altered proteostasis as part of OI’s pathogenic mechanism, treatment with the chemical chaperon 4-phenylbutyric acid (4-PBA) significantly reduced the intracellular retention of misfolded COL1 in murine osteoblasts, improving the physiological ER morphology and unfolded protein response markers and increasing collagen secretion and the mineralisation capacity. Similarly, in vivo 4-PBA treatment has shown promise in reducing ER stress, both in an OI zebrafish model and in human fibroblasts, through the improvement of protein secretion and the upregulation of the autophagic machinery [28,29,49]. Recent studies have proven the efficacy of ER stress inhibitor drugs in ameliorating OI’s pathogenic mechanisms, reducing the UPR and preventing apoptosis in patient-derived osteoblasts [50]. Additionally, the exogenous administration of the HSP47 chaperon enhanced proper COL1 folding and secretion, restoring the ER morphology and proteostasis and overall improving cellular viability [51].

### 2.2. Mitochondrial Stress

Common downstream effects of prolonged ER stress are mitochondrial dysfunction and redox imbalance [52]. The ER and mitochondria are highly dynamic organelles, interacting with each other both functionally and physically through mitochondrial-associated membranes, which are plastic, specialised contact sites created by the juxtaposition of endoplasmic reticulum membranes and mitochondria. Such contact sites play major roles in cell functioning, including the modulation of calcium signalling, lipid metabolism and cell survival/autophagy, and have also been identified as critical hubs potentially involved in the transmission of stress signals from the ER to mitochondria, specifically under conditions of disrupted proteostasis [53,54].

Therefore, considering altered collagen homeostasis and the associated ER stress, the presence of mitochondrial abnormalities is not unexpected in OI. Recent studies have evidenced the presence of mitochondrial dysfunction in several murine models: both oim/oim and heterozygous *Amish* mice display altered markers for mitochondrial mass/content and biogenesis and decreased mitochondrial respiration in skeletal muscles, which correlates with the muscle weakness reported in these mice [26,55]. Similarly, transcriptomic studies on *Amish* osteoblasts evidenced the disruption of ER–mitochondria contacts and overall increased mitochondrial distress, linked to increased misfolded procollagen accumulation in the ER [56]. In addition, changes in mitochondrial enzymes linked to oxidative and metabolic homeostasis (e.g., SOD2 and COX-IV), as well as changes in mitochondrial size, have been documented in muscle and cerebellar specimens from OI murine models [26,47].

Overall, mitochondrial defects, as reported in experimental models and human-derived samples, also contribute to altered energy metabolism in OI and may play a key role in the multisystemic symptoms of the disease, including muscle weakness, fatigue and impaired bone quality, through mechanisms involving metabolic imbalance and redox dysregulation [31,55].

### 2.3. Intracellular Responses to Homeostatic Imbalance

Beyond mitochondrial dysfunction, accumulating evidence regarding OI’s disease mechanisms points to alterations in autophagy and the activation of cell death mechanisms, including apoptosis, which reflect the cells’ attempts to cope with persistent stress and defective protein homeostasis. ER stress can activate autophagy as an adaptive response to remove damaged organelles and restore homeostasis, but, when the stress is severe or prolonged, apoptotic pathways are triggered through the loss of mitochondrial homeostasis, reactive oxygen species (ROS) accumulation and UPR-mediated proapoptotic signalling [38,57,58].

Misfolded collagen type I can accumulate in the endoplasmic reticulum, triggering response mechanisms designed to eliminate aberrant proteins, which include ER-associated degradation (ERAD) and autophagy. While ERAD specifically degrades misfolded soluble proteins via the proteasomal pathway, autophagy handles bulkier protein degradation as well as organelle clearance in a lysosomal-dependent manner [59,60]. In particular, in OI, mutant collagen is retained within cells, leading to increased autophagy: osteoblasts derived from *COL1A1* mutant mice display increased levels of lysosomal markers responsible for collagen degradation, suggesting the upregulation of autophagy, aimed at coping with aberrant collagen accumulation [30]. In vitro studies also demonstrate that autophagy serves as a major adaptive response to mutant procollagen accumulation. The in vitro administration of autophagy inhibitors increases the procollagen amount more prominently than proteasome inhibitors. Additionally, while autophagy inhibition increases collagen type I accumulation, enhancing the autophagic machinery improves cell stress in *COL1A2* mutant osteoblasts, suggesting a potential therapeutic intervention to mitigate OI pathology [25]. In 2023, Gosh et al. evidenced the upregulation of autophagy in fibroblasts with a mutation affecting the ER chaperon protein MESD, indicating that aberrant procollagen type I is degraded though autophagy and confirming the crucial role played by the autophagic machinery in OI proteotoxic stress [61]. Indeed, autophagy plays a key role in the intracellular dysfunction observed in OI. Mutant collagen misfolding activates the autophagic machinery; however, persistent protein accumulation can render this process insufficient, dysfunctional or overloaded. Consequently, in the presence of defective collagen accumulation, therapies aimed at restoring proper autophagic function appear promising in terms of improving cellular homeostasis.

Although autophagy may represent an attempted compensatory response, persistent and unresolved ER stress can ultimately culminate in the activation of cell death pathways; these mechanisms are also reflected in OI’s pathology, where the overmodification of collagen type I causes the increased activation of apoptosis in fibroblasts isolated from OI patients. Similarly, osteoblasts from the Aga2 mouse model display increased activation of caspase-12 and -3, as well as TUNEL positivity, demonstrating ER stress-mediated apoptosis and cell death as central features of OI’s pathophysiology [44,46]. Increased apoptosis in response to unresolved autophagy is also observed in fibroblasts carrying collagen type I mutations. Consistently, pharmacological modulation with the 4-PBA molecular chaperon, aimed at alleviating ER stress and restoring proper COL1 secretion, has shown promise in reducing apoptosis, enhancing proper autophagic degradation and overall improving cellular homeostasis [27,46,62].

### 2.4. Widespread Redox Imbalance and Inflammation

In osteogenesis imperfecta, persistent ER stress caused by misfolded collagen can disrupt mitochondrial function and overall alter the intracellular redox balance. The retention of unfolded/aberrant COL1 can be a major trigger for ER stress and the unfolded protein response (UPR), leading to the accumulation of ROS and resulting in the loss of intracellular homeostasis [38,63,64]. Impaired ER proteostasis, due to aberrant procollagen, in turn activates stress responses as a means to cope with ER stress, and their prolonged, unresolved activation leads compromised redox homeostasis and may culminate in cell death [25,65,66].

Given the ubiquitous expression of collagen type I and the activation of stress responses, cellular dysfunction in OI is not confined to bone-forming cells but may also affect other tissues, contributing to the multisystemic implications of OI, including muscle weakness, connective tissue fragility and cardiovascular or pulmonary alterations, which can manifest as widespread oxidative stress and inflammation with multiorgan and systemic involvement [13]. Altered levels of antioxidant and mitochondrial enzymes are reported in OI mice, indicating increased oxidative damage and integrated stress responses in diverse body regions [26,47,67]. Indeed, oxidative stress occurs locally but its effects can also spread, with cross-organ implications, through the activation of transcription factors that induce the release of circulating metabolites and proinflammatory molecules, leading to both local and systemic effects in distant organs [68,69,70,71]. Chronic redox imbalance over time can act as a driver of inflammation, and recent studies have evidenced a strong association with osteogenesis imperfecta’s pathophysiology and inflammatory processes, both in humans and in animal models [11,67,72]. Clinical reports indicate that patients with OI have an increased risk of developing inflammatory conditions such as glaucoma and inflammatory bowel disease [9,10,73]; additionally, co-occurrence with autoimmune diseases has been reported. Consistently, in vivo studies have shown elevated inflammatory markers, including TNF-α, IL-7 and TGF-β, in the bone marrow and peripheral blood of COL1 mutant mice, as well as in human patients; similarly, transcriptomic studies indicate the upregulation of interferon signalling pathways in blood samples from OI patients, overall indicating a chronic inflammatory state [16,67,74]. Increased proinflammatory conditions are likely to be both indirectly and directly linked to defective/aberrant COL1: on the one hand, recurrent fractures lead to frequent local inflammation, which can become systemic over time; on the other, alterations in the ECM can disrupt normal cellular signalling, predisposing patients to immune activation and dysregulation. Notably, collagen ligands include metalloproteases, adhesion molecules and cytokines; the extracellular accumulation of mutant COL1 can not only disrupt the immunological network (e.g., TGF-β signalling in oim mice) but might also contribute to autoimmune reactions [67,75,76]. Moreover, alterations in the hematopoietic and immune cell lineages have been reported in COL1 mutants, indicating strong proinflammatory conditions and supporting OI’s broader multiorgan implications [11,77,78].

Although further studies are still required to better define the extent and relevance of inflammation in OI, the recognition of widespread redox alterations and inflammatory features opens up new avenues for therapy, highlighting the potential of anti-inflammatory and immune-targeted approaches to complement existing bone-directed treatments [16,78].

## 3. Conclusions

Osteogenesis imperfecta is increasingly being recognised as a complex multisystem disorder, extending beyond structural collagen defects to involve cellular stress responses, redox alterations and chronic inflammation. The intracellular retention and misfolding of defective procollagen I chains can result in persistent ER stress and initiate a cascade of cellular stress responses, including ER stress, mitochondrial dysfunction and cell death, leading to chronic oxidative imbalance and inflammatory activation. Growing evidence from both human and in vivo/in vitro studies highlights that persistent ER stress, widespread homeostatic dysfunction and immune activation may exacerbate skeletal fragility, as well as contributing to the extraskeletal and systemic manifestations of OI.

Such findings broaden our understanding of OI’s pathophysiology and underscore the importance of multidisciplinary approaches that combine bone-targeting therapies with interventions addressing intracellular stress and inflammation. Antioxidant and anti-inflammatory/immunomodulatory strategies, either alone or in combination with conventional treatments, could represent promising avenues towards improving both skeletal and extraskeletal outcomes. Future studies should further clarify the molecular crosstalk between defective COL1, intracellular dysfunction, immune signalling and tissue homeostasis, both locally and systemically, with the aim of identifying more comprehensive and personalised therapeutic strategies. Progress in OI management will benefit from a deeper understanding of the disease’s complexity, encompassing multiple cellular stress pathways that may themselves represent potential targets for intervention, ultimately improving patients’ QoL.

## Figures and Tables

**Table 1:** Functional and metabolic grouping of osteogenesis imperfecta proposed by Forlino and Marini (2016) [6].

Group	Functional Defects	Genes
A	Collagen structure and processing	*COL1A1*, *COL1A2*, *BMP1*
B	Collagen modification	*CRTAP*, *LEPRE1*, *PPIB*, *TMEM38B*
C	Collagen folding and crosslinking	*SERPINH1*, *FKBP10*, *PLOD2*
D	Bone ossification and mineralisation	*IFITM5*, *SERPINF1*
E	Impaired osteoblast development	*WNT1*, *CREB3L1*, *SP7*

## Data Availability

No new data were created or analysed in this study. Data sharing is not applicable to this article.
